# Role of recombination and faithfulness to partner in sex chromosome degeneration

**DOI:** 10.1038/s41598-018-27219-1

**Published:** 2018-06-12

**Authors:** Dorota Mackiewicz, Piotr Posacki, Michał Burdukiewicz, Paweł Błażej

**Affiliations:** Department of Genomics, Faculty of Biotechnology, University of Wrocław, ul. Fryderyka Joliot-Curie 14a, 50-383 Wrocław, Poland

## Abstract

Sex determination in mammals is strongly linked to sex chromosomes. In most cases, females possess two copies of X chromosome while males have one X and one Y chromosome. It is assumed that these chromosomes originated from a pair of homologous autosomes, which diverged when recombination between them was suppressed. However, it is still debated why the sex chromosomes stopped recombining and how this process spread out over most part of the chromosomes. To study this problem, we developed a simulation model, in which the recombination rate between the sex chromosomes can freely evolve. We found that the suppression of recombination between the X and Y is spontaneous and proceeds very quickly during the evolution of population, which leads to the degeneration of the Y in males. Interestingly, the degeneration happens only when mating pairs are unfaithful. This evolutionary strategy purifies the X chromosome from defective alleles and leads to the larger number of females than males in the population. In consequence, the reproductive potential of the whole population increases. Our results imply that both the suppression of recombination and the degeneration of Y chromosome may be associated with reproductive strategy and favoured in polygamous populations with faithless mating partners.

## Introduction

The development of two sexually different organism is often associated in animals with various types of chromosomes. Generally, in mammals, males are heterogametic sex and possess sex chromosomes X and Y, while females are homogametic sex and possess two sex chromosomes X. However, there are a few exceptions, when females are XY and males XX^1^ or both sexes are X0^2,3^. It is commonly assumed that the sex chromosomes evolved from a pair of typical homologous autosomes^4–6^. The first step in the evolution of the Y chromosome was probably an acquisition of a sex-determining locus on one of proto-sex chromosomes^7^. After that, the suppression of recombination between X and Y was favoured to establish the position of this gene. The recombination could be suppressed by an inversion that encompassed the region with the sex-determining gene (SRY)^4,8^, which is responsible for determination of male sex^9^. Then, a selection favoured successive mutations and stepwise extension of the genetic linkage because it increased the probability of transmission of genes beneficial for one sex and detrimental for the other^10^. In consequence, the series of large-scale inversions happened on the Y chromosome and suppressed the recombination between the sex chromosomes almost completely^11^, which resulted in the accumulation of sexually antagonistic genes^12^. The recombination between the proto-X and proto-Y sex chromosomes could also be hindered by gradual reduction involving genetic modifiers^8,13^. The complete lack of the recombination led to a subsequent degradation and silencing of most Y-linked genes and created the male-specific region on the Y chromosome (MSY). Therefore, crossing over became restricted to only pseudoautosomal regions (PARs) on the Y chromosome^14,15^. However, it is worth to underline that the process of recombination in PARs between the X-Y chromosomes differs from that in autosomal chromosomes^16^. Although the sexual conflict model of sex chromosome evolution remains widely accepted, it is poorly supported by empirical evidence^17^. On the other hand, the sex chromosomes of many animals show evidence of strata, spatial clusters of X-Y orthologues with a similar divergence confirming past inversions^18,19^. However, we still cannot exclude that the recombination suppression was the contributory factor to inversions.

As a result of the isolation from recombination followed by degeneration, the human Y chromosome has lost during 170 million years hundreds of genes that were present on its ancestral autosome^20,21^. Currently, the Y contains only 78 genes on the 23 Mb^19^ and similar organisation of this chromosome is present in other vertebrates^22–27^. On the other hand, the X chromosome is comparable in size to autosomes and includes about 1,000 genes on the 180 Mb^28^. According to Ohno’s Law, X chromosomes contain a conserved gene content, which was inherited from their primordial X chromosome and changed very little during evolution^4^.

Despite the notable progress in the sequencing of Y chromosomes and identifying molecular mechanisms of sex determination, we still do not know exactly why and how sex chromosomes stopped recombining^17^. The issue of sex determination and sex chromosome evolution is still hotly debated^17,29–32^. It is also unclear, why the degeneration process of non-recombining sex chromosomes occurred and whether this process will continue or has already reached an equilibrium point^30^.

To explain the mechanisms of sex chromosome evolution, several theoretical models were proposed^33–37^. The first group of models focus on purifying selection against deleterious mutations and emphasize the role of genetic drift with “Muller’s ratchet”^38,39^. These models explain that each loss of the chromosome with the fewest number of deleterious mutations in finite populations cannot be restored in the absence of recombination^12,38,40,41^. In consequence, chromosomes carrying more mutations become fixed. On the other hand, the reduction of the effective population size of the Y decreases the efficiency of selection. It was proposed that the fixation of weakly deleterious mutations on a proto-Y chromosome was possible by a background selection leading to the degeneration of this chromosome in the long-term^42,43^. However, the theoretical models predict also that the acceptance of weakly deleterious mutations can be accelerated by the fixation of beneficial mutations on the Y in the process of genetic hitchhiking, when both types of mutations are linked^35,44,45^. Thus, evolutionary improvement at few loci can occur at the expense of most other genes on the Y chromosome.

A common feature of the theoretical models is the assumption that the efficacy of natural selection was strongly reduced on non-recombining chromosomes^39,46^. Hence, the chromosome-wide linkage resulted in the accumulation of many deleterious mutations and incorporation of fewer beneficial ones. In consequence, these models assume the lack of recombination and study its influence on the Y chromosome evolution^34,44^. Some of these models exclude recombination not only between the Y and X in males but also between the X chromosomes in females^33^. On the other hand, it was proposed that gene conversion allows ampliconic genes of the Y chromosomes to recombine, which could mimic the typical recombination between homologous autosomes^19,47^. This mechanism can explain surviving and expanding these genes on the Y chromosome during primate evolution, while many single-copy genes have decayed^37^.

The scenario described above convincingly explains that the suppression of recombination between the X-Y pair driven by sexually antagonistic genes could be assumed as the main reason responsible for the accumulation of deleterious mutations and the loss of genetic information in the Y chromosome. Thus, it can be conclude that the recombination between X chromosomes in females protects them from degradation^33^. Some simulations show that the increase in mutations on the Y depress male fitness, which initiates “demasculinization” of the Y and enables the recombination between X and Y in sex-reversed females^48^. However, the suppression of recombination between the X chromosomes does not cause their degeneration for two simulation models^33^. It suggests that an additional mechanism must exist to drive the accumulation of mutations on the Y. Moreover, some observations indicate that the sex chromosome divergence is not inevitable consequences of the recombination suppression^17^.

Since females have much greater impact on population reproduction than males and the X chromosomes spend two thirds of their evolutionary time in the females, the selection should favour the X chromosomes in the best possible genetic condition^33^. This suggests that the suppression of recombination between X and Y chromosomes as well as the degeneration of the Y can be associated with the role of females in population and a specific strategy of reproduction. Computer simulations demonstrated that the degeneration of Y chromosome occurs only if females find their new partner with every attempt of conception. When the female-male pairs are monogamous and faithful, the Y chromosome does not degenerate but evolves like the X, even if it does not recombine^49^.

In the absence of consensus about causes and mechanisms of degeneration of sex chromosomes, this subject needs further analyses. Therefore, we developed a more advanced simulation model to examine the evolution of sex chromosomes in terms of recombination and mating system. We simulated the evolution of a polygamous population, in which an individual of either sex may have more than one mate at the same time. We also considered a monogamous population, in which partners were faithful until the death of one of them. In contrast to the previous model^49^, we introduced the possibility of evolution of recombination between X and Y chromosomes to study its role in Y degeneration process. We also implemented a two-dimensional environment instead of the unrestricted environment with panmictic population to check the influence of spatial distribution of individuals on the studied phenomena.

## Methods

### Simulation model

The computer simulations were based on a modified Penna model^50^, which has been used many times and its usefulness was confirmed in many biological phenomena^51–56^. This model describes well the biological ageing process and introduces a link between the age and the genome^56^. Results of simulations using this model support mutation accumulation theory proposed by Medawar^57^ and they are in agreement with the empirical Gompertz law of an exponential increase in mortality with age. Moreover, this model well approximates the structure of real human populations^58,59^ and predicts well higher mortality of men than women in the middle ages as well as almost equal mortality for both sexes among the oldest groups of individuals after inclusion of sex chromosomes^60^. This fact was a motivation for using this model for our purpose.

**Figure 1:**
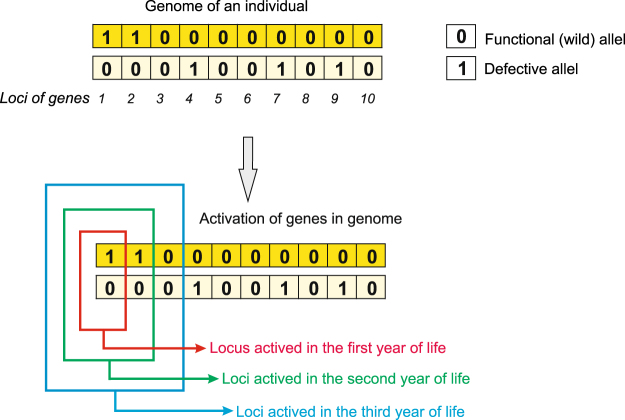
The example genome of an individual in the standard Penna model. The diploid genome is composed of two bitstrings corresponding to chromosomes, in which functional and defective alleles are marked by 0 and 1, respectively. The way of activating genes during the simulation is presented below. In the first year, two alleles in the first locus of the genome are activated. The phenotypic defect is expressed if the both alleles are mutated. In the second year, alleles in the second locus are activated and so on. The number of activated loci determines the age of the individual and increases with the simulation time. In the last year of individual’s life, all genes are active. Each individual can live as long as many bits are present in its chromosome.

The Penna model was initially designed for asexual populations with haploid genomes represented as single bitstrings^50^ but it was very quickly adopted for simulations of sexual populations^61,62^. In the sexual version of this model, the population is composed of diploid individuals. Each individual is represented by a diploid genome composed of two bitstrings which represent a pair of homologous chromosomes (Fig. 1). Bits correspond to genes. In the pair of bitstrings, two bits occupying the corresponding positions, i.e. locus, represent alleles responsible for the same function. The bit can equal 0, which corresponds to the functional wild type of allele, or 1, which corresponds to the defective (mutated) version of the allele. All defective alleles are recessive, which means that both alleles at a given locus have to be defective to determine the deleterious phenotype of that locus. We consider only harmful mutations because they are about 100 times more frequent in nature than the beneficial ones^63^.

The main assumption of the Penna model is the chronological activation of the pairs of alleles realized by the assumption that each bit (gene) corresponds to one “year” in the individual lifetime. It means that in each Monte Carlo step (MCs) of the simulation, the age of individual and the number of activated allele pairs are increased by one (Fig. 1). Therefore, one simulation step corresponds to a time unit/period. Each individual reaches its maximum age when the whole genome is activated. The age corresponds to the length of the chromosome *L*. Thanks to that the Penna model describes the population age structure and individuals can die because of the old age besides the accumulated mutations. The gene activation is realized by checking a given locus whether it causes a deleterious phenotype. In each simulation step, the number of defective loci activated till the age of a given individual is counted (Fig. 1). When the number reaches the assumed threshold *T*, the individual dies because of genetic defects. The age-dependent accumulation and disclosure of mutations is in concordance with the fact that the number of mutations increase with age^64^ and many diseases become apparent only in old ages, e.g. Huntington’s, Parkinson’s or Alzheimer’s diseases, although corresponding defective genes occur in the genome before the diseases are revealed. Thus, this model simulates the case in which many mutations being neutral for young individuals can be passively accumulating over generations leading to senescence and death because of deleterious effects^65^. Accordingly, there are hundreds of age-regulated genes in animals including humans^66–69^, which may serve as markers of aging and allow to assess physiological age^70^. Collectively, mutations in some genes cause dramatic changes in lifespan^71^.

**Figure 2:**
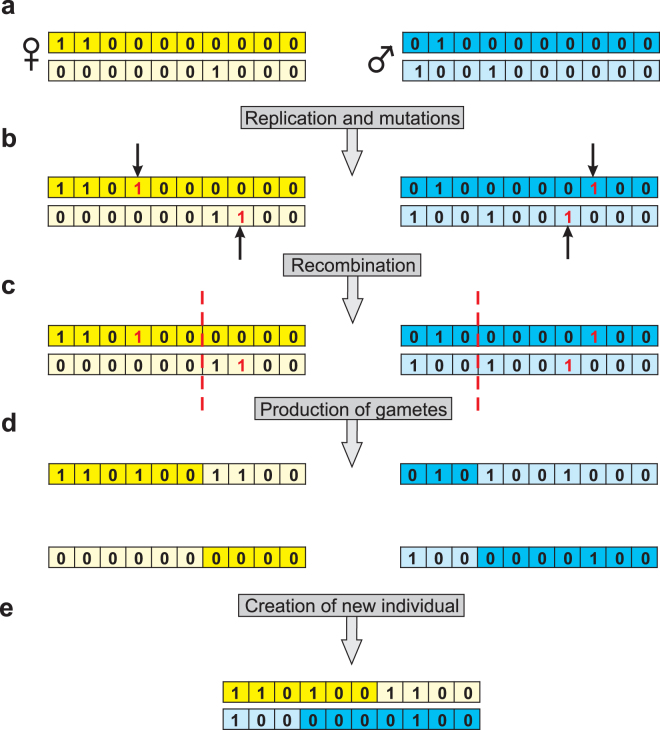
The stages of generation of a new individual. (**a)** Two diploid parental genomes represented by pair of bitstrings, i.e. homologous chromosomes, participate in the generation of a new offspring. (**b)** The genomes are duplicated to imitate DNA replication process. During this process, a new mutation, marked by an arrow, is introduced with probability *M* into a randomly chosen bit, i.e. gene, on the replicated chromosomes. (**c)** Next, during the formation of gametes, the new copies of bitstrings recombine with probability *C* at the randomly chosen intergenic sites, marked by red dashed lines. (**d)** The process produces haploid gametes. (**e)** One of two possible gametes is chosen from each of partner and a diploid zygote is formed.

When an individual reaches the minimum reproductive age *R*, it can reproduce every year until its death. The number of conception attempts in a given MCs is randomly chosen from Poisson distribution with the average *B*. Genomes of new-borns are constructed in the reproduction process mimicking meiosis with gamete production and fertilization (Fig. 2). Each female in the reproductive age attempts to conceive with a male. A female can choose different males for every attempt of conception, and a male can be selected by several females in one simulation step. The genome of each partner is replicated and during this process a new mutations can be introduced into each chromosome at the randomly chosen position with a probability *M*. Thus, *M* is the average number of mutations per chromosome. Mutations are realized by the change of bit from 0 to 1. When a bit chosen for mutation is already mutated, its label remains unchanged. Therefore, there are no reversions. Next, the two reciprocal bitstrings recombine with a probability *C* at randomly chosen positions. One of the two products of the crossover is randomly chosen as a gamete. The female and male gametes form the diploid genome of an offspring. Next, the first locus of each chromosome is checked for the genetic status, if it is mutated or not.

**Figure 3:**
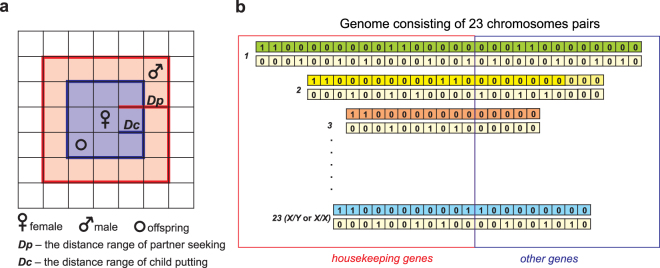
Modifications of the standard model. (**a)** A part of lattice with the position of a female seeking for her potential partner in the distance range *D*_*p*_ and putting their child in the distance range *D*_*c*_. (**b)** The simplified genome scheme composed of 23 pairs of chromosomes used in the study. One group of genes, called housekeeping, is activated at once since the embryo development. The second smaller group of genes is activated only chronologically during the lifetime of individual.

We introduced several modifications into the classical Penna model for the purpose of our simulation study. In our model, the population evolves on a square lattice representing its environment (Fig. 3a). The lattice is evenly divided into 128 × 128 cells and its size determines the maximum number of individuals in the population because each cell can be occupied by only one individual. The lattice did not suffer from boundary condition because its opposite edges are joined. In fact, the lattice is a torus.

In the standard version of the Penna model, the environment limits are described by the Verhulst factor^50^, which controls the growth of the population size. However, this approach was criticised because this factor seems too robust to reconstruct real dependencies existing in biological systems^72,73^. In contrast to that, the Penna model based on the lattice describes better the mortality curves of human population^74^. Therefore, we applied the lattice model.

Moreover, we used features characterizing the real human genome to create the simulated genomes. We included 23 pairs of chromosomes with the number of genes matching real values. The number of genes was taken from the Ensembl database (version 57) (http://www.ensembl.org/index.html). Each individual had 23 pairs of bitstrings with the number of genes rounded to the multiples of 128, which was the maximum life-span and the basic chromosome length in our simulations (Table S1). One pair of chromosomes was defined as sex chromosomes. In males one chromosome was marked as Y and the other as X, whereas in females the both were marked as X. The sex chromosomes were identical at the beginning of simulations. The number of activated genes was proportional to the length of chromosomes and was equal to multiplier of the basic chromosome length, i.e. 128. For example, if a chromosome contained 7 · 128 = 896 bits (genes), we activated 7 genes in one simulation step. Among the activated genes, we counted those whose both alleles were mutated and we added this number to the number of defective genes already accumulated in previous simulation steps. The sum was compared with the threshold *T*.

We also introduced the mutation rate proportional to the length of chromosomes (Table S1) in order to the average rate was equal to one per genome per generation, which corresponds to the estimated mutation rate in human genome falling within the range 0.9–4.5^75–79^. The assumed value agrees also with Azbel’s calculations showing that the optimal rate of mutations should be about one mutation per genome per generation, independently of the size of the genome^80^. Many experimental results have shown that at least the order of this estimation is correct^75^. The assumed number of recombination events *C* is based on the genetic maps for human chromosomes^81^. It is worth to underline that the probability of recombination and mutation are different for each pair of chromosomes (Table S1). The values of mutation are based on our previous analyses^82^.

In our model, the attempt of conception consisted of: (1) looking for a partner being also in the reproductive age in the distance range *D*_*p*_ on the lattice, (2) looking for a free place on the lattice to put a child in the distance range *D*_*c*_, (3) mutations and recombination realized during the production of gametes, (4) combining gametes and (5) checking genes for defective phenotype, activated before the birth age *b*. The last stage is also an innovation in comparison to the standard Penna model and assumes the substantial number of so-called housekeeping genes (Fig. 3b) that are activated at once since the embryo development^72,83^. When the number of defective loci of these early activated genes exceeds threshold *T*, the embryo does not survive. Therefore, this assumption can model a high probability of zygotic death, which is actually observed in nature^84,85^. The creation a new individual is successfully realized when the number of defective homozygous loci is lower than *T* and a free place on the lattice in the distance range *D*_*c*_ is found. Females and males have the same probability of birth.

Moreover, we modified the standard Penna model including: (1) the possibility of recombination between X and Y chromosomes; normally only X chromosomes in female and autosomes in both sexes recombine, (2) the evolution of X-Y recombination during simulation by inheritance of father’s X-Y recombination value by sons, (3) the possibility of change of reproduction strategy, in which individuals create a stable and faithful female-male pairs until one partner dies. In the case, when the X-Y recombination can evolve, the initial value is set up to the recombination value between X chromosomes. The inherited X-Y recombination value could change by +/− 0.05. The decrease in the recombination to 0 caused the stopping the evolution of recombination.

We performed several types of simulations resulting from all possible combinations of: (1) two different strategy of reproduction, with and without faithfulness of mating pairs, and (2) three assumptions on recombination between the sex chromosomes: no recombination, constant recombination and evolving recombination during simulations.

The simulations started with the lattice filled up with individuals of randomly chosen sex and age with no mutations, and were carried out for 1,000,000 Monte Carlo steps. Each type of simulation was conducted 50 times and average values of studied free parameters were presented in the paper. Results obtained for individual repetitions were very similar and showed very small variation. Thus, the increase of repetitions’ number would not influence our conclusions. Values of main parameters used in the simulations were as follows: the basic chromosome length *L* = 128; the number of loci checked before birth *b* = 75; the reproductive age *R* = 105; the number of homozygous loci causing genetic death of individual *T* = 3 for simulations with unfaithful pairs or *T* = 20 for simulations with faithful pairs; the distance range of partner seeking in the lattice *D*_*p*_ = 6; the distance range of child putting in the lattice *D*_*c*_ = 6; the average number of attempts of conception per female per simulation step *B* = 8. If the conception was successful, the female did not make other attempts.

Analysing results, we considered separately three groups of individuals depending on their age: new-borns, youths and adults. The group of new-borns includes individuals at the birth time. The group of youths contains individuals between the birth time and the age of reproduction, while the group of adults comprises individuals in the reproductive age.

### Calculation of chromosome divergence

To study the divergence of sex chromosomes and the 10th autosome, we compared the corresponding sequences of human chromosomes with those from chimpanzee (*Pan troglodytes*), gorilla (*Gorilla gorilla*) and rhesus macaque (*Macaca mulatta*). The 10th autosome was used as a reference to chromosome X because it has comparable number of genes and the value of recombination as the X. The sequences were downloaded from Ensembl database release 90^86^ as the following assemblies: human GRCh38.p10, chimpanzee CHIMP2.1.4, macaque Mmul_8.0.1 and gorilla Gor3.1 for X and the 10th chromosomes, whereas the newer assembly of gorilla Y was downloaded from NCBI database as GCA_001484535.2_GorgorY_ver1.0. The homologous non-overlapping one-to-one mapping regions of chromosomes were found using MUMmer^87^.

Moreover, we compared orthologous gene sequences of human and chimpanzee located on corresponding chromosomes. The sequences of orthologous were downloaded from Ensembl database by using BioMart tool^88^. We have analysed only the best hit one to one orthologous. The sequences were aligned using stretcher from EMBOSS package^89^. The stretcher calculates optimal global alignment of two sequences using a modification of the classic dynamic programming algorithm by Needleman-Wunsch^90^. The fraction of different nucleotides between the aligned sequences was calculated using ClustalW^91^.

The divergence between chromosome region and genes was corrected for multiple substitutions using the formula of Jukes and Cantor^92^. The differences in the divergence of regions or genes between different chromosomes were assessed using Dunn Kruskal-Wallis test for multiple comparison and p-values were adjusted with the Benjamini-Hochberg method, as implemented in R package^93^.

## Results

### Evolution of population with and without recombination between X and Y chromosomes

To evaluate the role of recombination between X and Y chromosomes in evolution of population, we carried out simulations with switched on and switched off recombination. There were no restrictions for mating, as in the standard Penna model, i.e. females could mate with many males and *vice versa* if the partners were in the distance range *D*_*p*_ = 6. Their child was put on the lattice in the same distance range *D*_*c*_ = 6. We analysed the distribution of fraction of defective genes in respect of their activation time for the last step of simulations as well as compared the ratio of defective alleles in chromosomes and the females to males ratio during the simulation time. The latter results were recorded for three groups of individuals depending on their age: new-borns, youths and adults.

**Figure 4:**
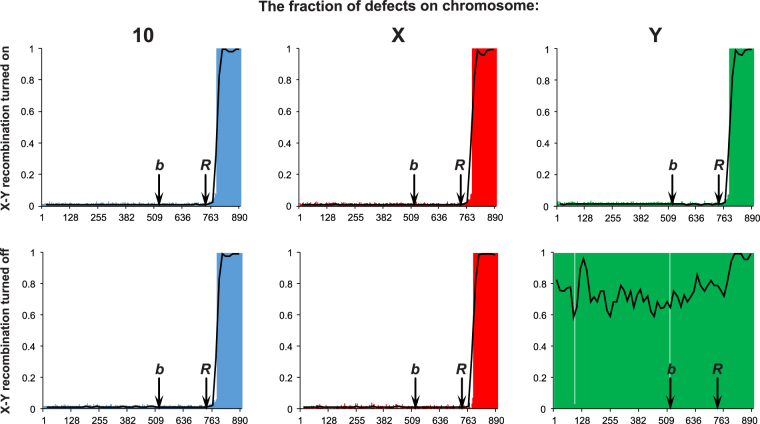
Fraction of defective alleles in the loci of the 10th autosome or sex chromosomes calculated in all individuals after 1,000,000 MCs. The x axis corresponds to genes ranked according to their activation time; the y axis corresponds to the mean fraction of deleterious alleles for the given gene; *b* is the time of birth; *R* is the first year of reproduction. The upper plots concern the simulations with turned on recombination between the X and Y chromosomes. Note, the growing fraction of defective loci activated after the minimum reproductive age. The bottom plots refer to the simulations with turned off recombination between the X and Y chromosomes. Note, the large fraction of defective loci on the Y chromosome independently on the activation time of genes.

**Figure 5:**
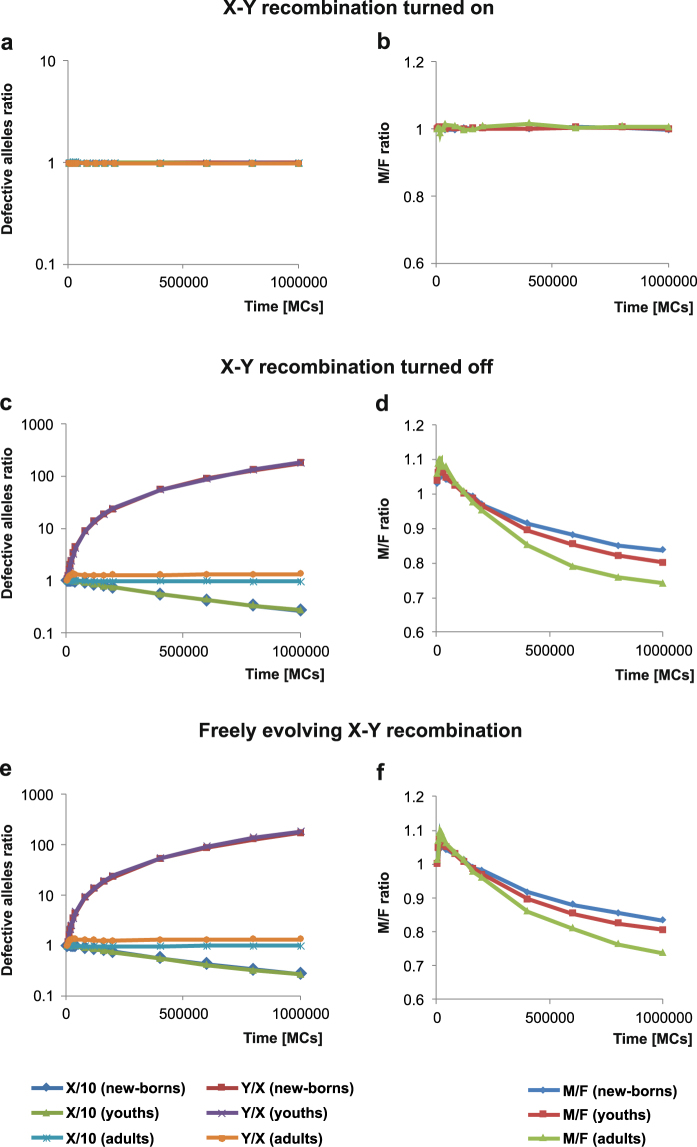
Results of computer simulations with unfaithful mating pairs for three assumptions on the recombination between X and Y chromosomes. The left panel (**a**,**c**,**e**) The ratio of the number of mutated alleles in the X chromosome to the 10th autosome (X/10) and in the Y chromosome to the X chromosome (Y/X), calculated for individuals in various age: new-borns, youths and adults. The right panel (**b**,**d**,**f**) The ratio of males to females (M/F) calculated for individuals in various age: new-borns, youths and adults. New-borns are individuals before the birth age; youths are individuals between the birth and reproductive age, and adults are individuals after the reproductive age. Points for the ratio of defective mutations in Y/X (new-borns) and Y/X (youths) as well as for X/10 (new-borns) and X/10 (youths) overlap.

In the simulations with allowed recombination between X and Y chromosomes and the recombination value equal to that between X and X, we received the characteristic distribution of fraction of defective genes (Fig. 4). The genes that were activated before the minimum reproductive age *R* rarely accumulated mutations because of strong selection. On the other hand, the genes activated after the reproductive age accumulated many defective alleles whose fraction reached 100% in the genes activated at the latest in the life of individuals. These genes were subjected to much weaker selection because started to play a role for surviving individuals only in the late periods of their life. It is worth to notice that there are no differences in the mutation distribution on autosomes and the sex chromosomes (Fig. 4). The sex chromosomes X and Y accumulated a similar number of mutations like the 10th autosome, which was chosen as a reference for the sex chromosomes because it has the comparable number of genes and the value of recombination as the X. The lack of differences in the number of accumulated mutations between the chromosomes is well visible in the ratio of accumulated defective mutations, X/10 or Y/X, which is equal to 1 (Fig. 5a). There are no difference in this ratio for three age groups either. The ratio of males to females also did not depend on the age of individuals and remained close to 1 during the whole simulation (Fig. 5b). These results imply that the unrestricted recombination between X and Y chromosomes keeps similarity in the evolution of sex chromosomes and autosomes.

However, results changed significantly when the recombination between the X and Y chromosomes was forbidden. The substantial rise in the number of defective alleles in the genes activated after the reproductive age was maintained in the X and autosomes but genes in the Y chromosome accumulated mutations independently on their activation time. As a result, the whole chromosome was almost evenly covered by mutated alleles (Fig. 4). The Y chromosomes are also characterized by the sudden increase in the fraction of mutated alleles during simulation time in comparison to the X chromosome (Fig. 5c). This increase is the largest for new-borns and youths, for which the number of deleterious mutations is about 180 times greater in Y than in X at the end of simulations. This is due to the fact that the X chromosome accumulates much less mutations than the Y. The ratio of defective mutations in Y to X shows almost a linear relationship with the simulation time (R^2^ = 0.98) and the slope is 16.69 per 100,000 MCs for new-borns and 16.88 per 100,000 MCs for youth. It indicates a linear accumulation of mutations in the Y compared with the X chromosome. However, the Y/X ratio for adults increases to only 1.35, which results from a more intense accumulation of the defective mutations in X and saturation in the number of mutations in Y since the reproduction time.

Because of the stronger selection against accumulation of mutations in chromosome X, the number of defective genes in the X in relation to the 10th autosome decreases during the simulation time and is about 0.27 at the end of the simulation for new-borns and youths while for adults is about 0.99 (Fig. 5c). The reduction of defective mutations on the X chromosomes is associated with the better fitness of females than males. In consequence, the number of females (F) in population is much larger than males (M), especially among the oldest group of individuals in mature populations. Therefore, the M/F ratio is generally below one (Fig. 5d). It should be noted that we did not apply any preference for male or female birth.

The relations of mutations accumulated during simulations in the 10th, X and Y chromosomes (Fig. 5c) agrees with the relations of substitutions accumulated in homologous regions or genes of corresponding chromosomes since the split of human and other primates: chimpanzee, gorilla and macaque (Table 1, Figure S1). The number of accumulated mutations in the 10th autosome is always larger than in the X chromosome, from 1.1 to 1.7 times on average. Much greater difference is between the Y and X chromosome; the former accumulates from 1.7 to 3.8 times more mutations on average. The differences in the divergence values for each pairwise comparison of chromosomes are statistically significant with p-value < 0.016. The divergences calculated for orthologous genes located on the three chromosomes form separate distributions (Figure S1).

**Table 1:** Divergence between homologous regions or genes of corresponding chromosomes between human (H) and: chimpanzee (C), gorilla (G) and macaque (M).

Type of comparison	Chromosome 10		Chromosome X		Chromosome Y		Ratio of medians	
	Med. [quart. range]	N	Med. [quart. range]	N	Med. [quart. range]	N	X/10	Y/X
H-C (regions)	0.021 [0.017–0.029]	6272	0.016 [0.012–0.022]	18455	0.038 [0.024–0.120]	601	0.757	2.356
H-G (regions)	0.025 [0.020–0.032]	13388	0.022 [0.017–0.029]	15279	0.083 [0.038–0.148]	1288	0.875	3.829
H-M (regions)	0.133 [0.104–0.160]	9717	0.079 [0.068–0.095]	17391	0.137 [0.119–0.161]	1907	0.593	1.738
H-C (genes)	0.012 [0.010–0.015]	617	0.009 [0.007–0.013]	548	0.015 [0.014–0.015]	12	0.759	1.670

Med. - median; quart. - quartile; N - number of pairs of compared regions or genes.

### Evolution of population when the recombination between X and Y chromosomes can freely change

**Figure 6:**
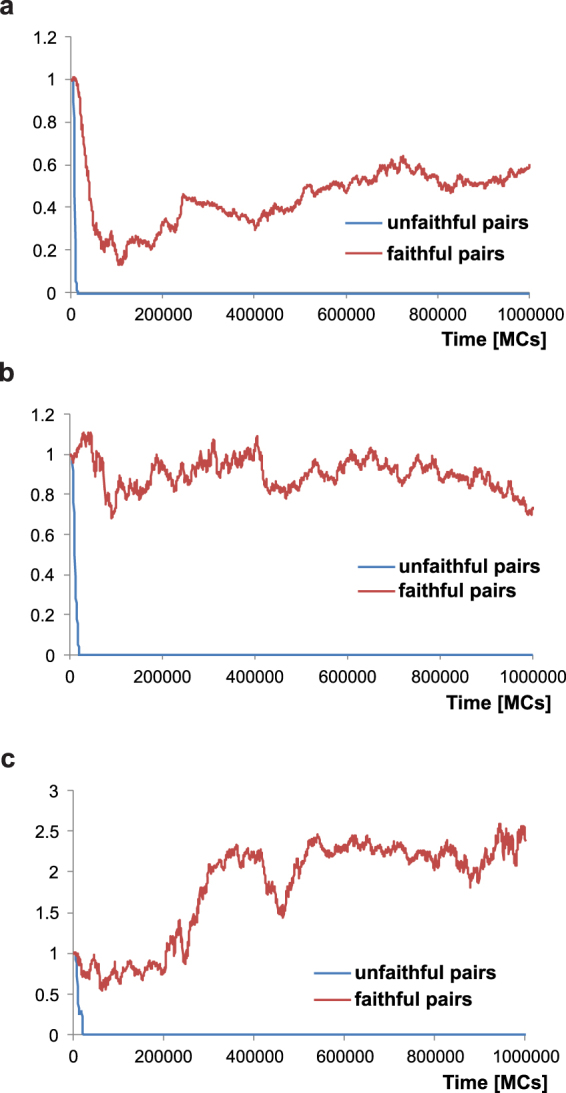
Changes in the recombination rate between the X and Y chromosomes during simulations for two reproductive strategies: unfaithful and faithful mating pairs. Values on the y axis are mean of recombination in a given simulation step divided by the initial value typical of X chromosomes. Three models were considered, with the lattice assuming distance ranges for searching for a partner and putting a child, *D*_*p*_ = 6 and *D*_*c*_ = 6 (**a**), *D*_*p*_ = 64 and *D*_*c*_ = 64 (**b**) as well as a model without the lattice (**c**).

In other type of simulations, we allowed the recombination between chromosomes X and Y to evolve freely since the beginning of the simulations. The initial recombination rate was typical of chromosomes X in females. Figure 6 shows the evolution of this recombination in relation to the initial value typical of X chromosomes.

In the case of simulations with unfaithful mating pairs, the recombination between X and Y chromosomes turned off very quickly. The initial recombination rate stayed unchanged for only very short time and then rapidly decreased to 0 after 17,000 MC steps (Fig. 6a). Therefore, the results were very similar to those in the simulations without allowed X-Y recombination. The Y chromosome degenerated and the X was purified by selection in comparison to the 10th autosome (Fig. 5e). The mutation ratio Y/X showed also a linear relationship with the simulation time (R^2^ = 0.98) and the slope was 16.41 per 100,000 MCs for new-borns and 17.56 per 100,000 MCs for youth. After a short period of male preponderance, females became more numerous (Fig. 5f), which was associated with the accumulation of defective alleles on the Y. The results imply that the short time with allowed X-Y recombination at the beginning of the simulations was not enough to stop the degeneration of Y.

Since the population in our model evolves in a lattice with a fixed distance range for partner search (*D*_*p*_ = 6) and child putting on the lattice (*D*_*c*_ = 6), we checked the influence of the environment restriction on the results. Therefore, we ran two additional types of simulations. Firstly, we let the individuals search for a partner and put a child on the whole lattice without restrictions, assuming large respective distance ranges, *D*_*p*_ = 64 and *D*_*c*_ = 64. Moreover, we resigned from the lattice and simulated the panmictic population in the Penna model as other authors^33,49^. However, these models limited the population size by Verhulst factor as well as assumed one pair of shorter sex chromosomes and at most one pair of autosomes. Nevertheless, we did not observe substantial differences between our simulation types. The turning off recombination appeared in 24,000 MCs for the first simulation type and in 22,500 MCs for the second type (Fig. 6b,c).

As so far, we observed that the lack of X-Y recombination leads to the degeneration of Y, which is associated with the decrease in the number of males in population. This strategy seems favoured in population when females are more important for survival of the population than males and when the females are promiscuous and seduce the males^49^. Therefore, to check if the stopping recombination between X-Y chromosomes is sufficient to induce the degeneration of the Y chromosome, we included faithful mating pairs in simulations. Under this scenario, there was no differentiation in the mutation pattern between sex chromosomes in comparison to autosomes, regardless of X-Y recombination (Figure S2a,c). The X/10 and Y/X ratios were close to one for the whole simulation time. The lack of accumulation of mutations on the Y chromosome was associated with the male to female ratio, which was also close to 1 (Figure S2c,d).

We assumed initially the limit of mutations responsible for genetic death *T* = 3 as other authors^33,49^. However, to keep the population with faithful pairs alive up to 1,000,000 MCs, it was necessary to loosen selection constraints by increasing *T*, from 3 to 20. To check if this change has an influence on results, we also repeated all simulations for unfaithful pairs with the same threshold *T* = 20 (Figure S3–S5). Nevertheless, we observed no influence on the final conclusions about the suppression of recombination and the degeneration of Y chromosome. There were no important qualitative differences compared with the simulations assuming *T* = 3. Only fraction of accumulated defects was slightly higher for *T* = 20.

Finally, we let the X-Y recombination freely evolve in the simulations assuming faithfulness of mating pairs. In this case, we did not observe suppression of the recombination process. The rate of recombination decreased sharply at the beginning of simulation and then raised to remain stable around 0.5–0.6 value up to the end of simulations (Fig. 6a). We did not observe the degeneration of Y chromosome (Figure S2e) and the M/F ratio was also nearly equal to one (Figure S2f). The results did not change for the simulations without the lattice (Fig. 6c) as well as for those with the larger distance range for a partner seeking and a child putting on the lattice (Fig. 6b).

**Figure 7:**
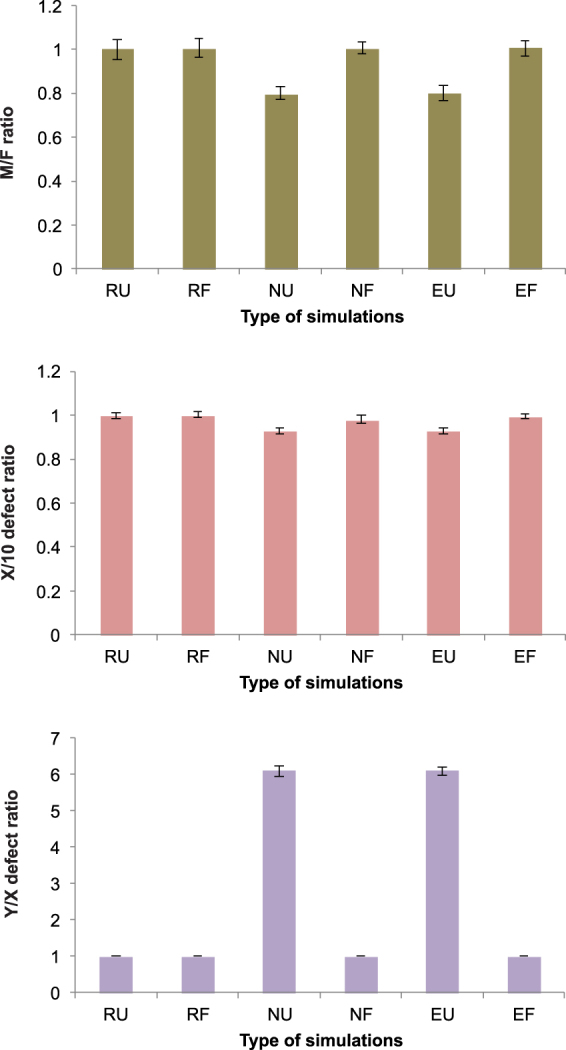
The comparison of the average minimum and maximum values of males to females ratio (M/F) as well as the ratio of the number of mutated alleles in in the X chromosome to the 10th autosome (X/10) and in the Y chromosome to the X chromosome (Y/X) for different simulation scenarios. The average values were calculate for 50 repetitions of simulations in each scenario after 1,000,000 MCs. Various parameters and conditions of the simulations are marked by abbreviations. T is the threshold for the number of defective loci which kill the individual because of the genetic death. The parameters and conditions of simulations: RU - X-Y recombination turned on and unfaithful pairs; T = 3, RF - X-Y recombination turned on and faithful pairs; T = 20, NU - X-Y recombination turned off and unfaithful pairs; T = 3, NF - X-Y recombination turned off and faithful pairs; T = 20, EU - freely evolving X-Y recombination and unfaithful pairs; T = 3, EF - freely evolving X-Y recombination and faithful pairs; T = 20.

The final results are summarized in Fig. 7, where we collected data from the 1,000,000th MCs for six different scenarios of simulations. The data demonstrate that the reduced Y chromosomes and the biased sex ratio can evolve only in the case of unfaithful mating pairs under turned off X-Y recombination. On the other hand, the faithfulness of mating pairs protects chromosome Y from degeneration in each version of simulation. Concluding, the accumulation of deleterious mutations on the Y and in consequence diminishing the number of males as well as the selection against the accumulation of defective mutations on the X chromosome is under the influence of the reproductive strategy.

## Discussion

Factors and mechanisms of the stopping recombination between X and Y chromosomes are still controversial^17^. Previous models focused rather on the Y degradation after switching off the recombination^33–35^, tried to check the effect of shrinking the Y chromosome on its survival^37^, or find processes which protect against the degeneration after suppression of recombination^48^. Collectively, these models assumed only a panmictic population and including only sex chromosomes^34^ or even only the Y chromosome^35,37^. Therefore, besides the X and Y chromosomes, we also included 22 pairs of autosomes to imitate a diploid human genome.

Moreover, we applied the aging structure in order to our model was comparable with others which also included the aging^33,49^. Although, at the first look, it may seem that the Penna model is not necessary to study the degeneration of the Y chromosome, it can be important in the analysis of the recombination evolution. This is because the model with aging allows to include the beneficial role of recombination in purifying chromosomes from defective alleles which can accumulate with age of individuals. Furthermore, this model is also justified by biological studies that showed that age could be a significant factor associated with the recombination process^94–96^. Nevertheless, it is interesting to study the subject using models with various parameters and other assumptions.

We introduced a model with a lattice and the distance-dependent searching for a partner, which seems be a better approach to describe the real environmental limitations on the population growth and structure than the commonly used Verhulst factor^74^. And the most important, we checked conditions with or without X-Y recombination and allowed the recombination to freely evolve.

When the recombination was enabled throughout the entire simulation, there were no differences in the number of accumulated mutations between the sex chromosomes and autosomes, sex chromosome ratio as well as the number of males and females. The results were the same for the populations both with unfaithful or faithful partners. When the recombination was disabled, the Y degenerated and the number of females exceeded the number of males but only when the mating pairs were unfaithful. In the case of faithful partners, the Y chromosome preserved all genetic information and the biased sex ratio was not observed.

To study if the reproductive strategy can cause the cessation of the recombination, which is not possible in the simulations with the turned off or on X-Y recombination, we let the recombination rate evolve freely and self-organize during the simulations depending on benefits for the population. The results showed that the cessation of the recombination is a spontaneous and rapid process but only in populations with unfaithful mating pairs. It is worth to notice, that the results were the same when we increased the distance range of looking for a partner to 64 or resigned from the lattice.

Our simulations show, that there is no need to introduce neither inversions nor additional mechanisms to stop the recombination between X-Y because the unfaithful reproductive strategy can be sufficient to initiates this process and start the accumulation of mutations on the Y chromosome. It also consistent with several studies of nascent sex chromosomes suggesting that the recombination suppression was initially a heterogeneous process^97–99^. As a result of the suppression of the X-Y recombination, the Y chromosome degenerates, whereas the X chromosomes are subjected to strong purifying selection against defective alleles because males have only one copy of the X and this chromosome has to complement mutations exposed in the degenerated Y. Due to the Y decay, only males possessing a better purified X have a higher probability of survival. The elimination of deleterious mutations on the X increases the fitness of females at the expense of males. In consequence, the number of females in the reproductive age grows, which finally improves the population reproductive potential because females bear costs of offspring birth and their role in the population is regarded more important than males. It is evident in polygamous populations with unfaithful mating pairs. Then, the lower number of males is sufficient to keep the reproductive potential of population because many females can mate with the same male. As a result of this strategy, the recombination between X-Y is suppressed, the Y degenerates and mortality of males increases compared with females. This strategy is beneficial for a reproductive potential of population because females do not have to compete with large number of males for resources^100–102^.

The reproductive strategy involving unfaithful partners seems very common in mammals. Only 3–5% out of 4000 mammalian species live in monogamy^103^. It is claimed that humans were essentially polygynous during much of their history^104^. Even among animals considered monogamous^103,105,106^ as well as humans^107–110^, it is quite common that partners are not completely faithful because of ‘genetic promiscuity’. It is known that internal gestation and lactation in mammals are essential components of parental care limited to females. Consequently, the number of offspring per a female is much smaller than per a polygamous male^111^. Therefore, it is not clear why monogamy occurs in some animal species as a reproductive strategy because it leads to the loss of male reproductive potential^112,113^. In the case of human, social monogamy has likely evolved under cultural factors, particularly religion^103^.

The results of our simulations with unfaithful partners agrees well with empirical comparison of human and chimpanzee genomes demonstrating that the mean divergence of the Y chromosome is significantly higher than the X as well as that the divergence of the X is lower than that of autosomes^114–117^. Our calculations of the divergence between homologous regions or genes of corresponding chromosomes between human and three primate representatives, also showed the differential substitution accumulation on the chromosomes in various evolutionary scales (Table 1). Interestingly, we obtained the similar fractions of accumulated mutations in the simulations without *a priori* assumption on the different substitution rate on chromosomes, i.e. 6.097 for Y/X and 0.928 for X/10. However, the accumulation of defective alleles on the Y and their eradication from the X occurred when recombination between these chromosomes was turned off and the population was polygamous. No differences in the mutation accumulation were observed when monogamy was a reproductive strategy of the population. It indicates that the differentiated accumulation of mutations on chromosomes can be associated with the reproductive strategy, not only with a higher mutation rate in the male than female germ line, which is explained by higher number of germ cell divisions in males than females^118,119^.

On the other hand, our simulations demonstrated that the X-Y recombination is not suppressed in the monogamous population consisting of faithfully mating pairs. In such population, males and females are both necessary for survival of offspring and the highest reproductive potential is gained when the males to females ratio equals 1. Therefore, mutations on the Y chromosome become much more disadvantageous than in the population with unfaithful pairs. The accumulated mutations on the Y would reduce substantially the reproductive potential of the whole population because of greater mortality of males whose role is as important as females in the monogamous populations. That is why the suppression of recombination is not favoured under such reproductive strategy and then monogamy can explain why the recombination continues to happen and did not stop. There are also other reasons why the recombination did not stop completely in nature. The time that has elapsed since the evolution of Y chromosome could be too short to turn off the recombination ultimately^30^. Moreover, the recombination is kept because at least one crossover per chromosome arm is necessary to correctly segregate the chromosomes during meiosis to daughter cells^120^.

The importance of faithfulness seems to override the influence of recombination on the evolution of sex chromosomes because the Y chromosome did not decay both under the presence and the absence of X-Y recombination when females are not promiscuous. The significance of the reproductive strategy implies that inversions and sexually antagonistic loci could occur on the Y chromosome after recombination suppression, which could be further supported by polygyny because of lowering the Y effective pool size. In consequence, the loss of functional genes on the Y caused the evolution of gene dosage compensation to balance an unequal gene expression between sexes. This process is almost complete in mammals^121,122^.

Finally, the presented results can mean that many mechanisms were involved in the evolution of sex chromosomes. Thus, it is cannot be ruled out that both the strategy of reproduction and the accumulation of sexually antagonistic loci together with inversions hasten the degeneration of Y chromosome because the recombination between X and Y chromosomes is not profitable in these cases. Investigations of organisms with sex chromosomes being in the early stages of degeneration can help to solve the discussed problems.

## Electronic supplementary material

Supplementary material
