# Supplementary material for: Role of recombination and faithfulness to partner in sex chromosome degeneration
Source: Sci Rep. 2018 Jun 12;8:8978. doi: 10.1038/s41598-018-27219-1 (PMC5997740; doi:10.1038/s41598-018-27219-1)

Supplementary Information

Role of recombination and faithfulness to partner in sex chromosome degeneration

Dorota Mackiewicz*, Piotr Posacki, Michał Burdukiewicz, Paweł Błażej

Department of Genomics, Faculty of Biotechnology, University of Wrocław, ul. Fryderyka Joliot-Curie 14a, 50-383 Wrocław, Poland.

Correspondence should be addressed to D.M. (email: dorota@smorfland.uni.wroc.pl)

Table S1. Parameters applied for particular chromosomes in simulations.

Chromosome	Chromosome length (*L*)	Recombination events (*C*)	Probability of mutation (*M*)
1	2176	2.7	0.097
2	1408	2.58	0.063
3	1152	2.18	0.051
4	896	2.02	0.040
5	896	2.05	0.040
6	1408	1.9	0.063
7	1152	1.79	0.051
8	768	1.59	0.034
9	896	1.57	0.040
10	896	1.76	0.040
11	1408	1.52	0.063
12	1152	1.71	0.051
13	384	1.28	0.017
14	768	1.18	0.034
15	768	1.29	0.034
16	896	1.28	0.040
17	1280	1.35	0.057
18	256	2.1	0.011
19	1408	1.09	0.063
20	640	0.98	0.029
21	256	0.72	0.011
22	640	0.65	0.029
X	896	1.79	0.040

Figure S1. Distribution of divergence calculated for orthologous genes located on three chromosomes in human and chimpanzee.

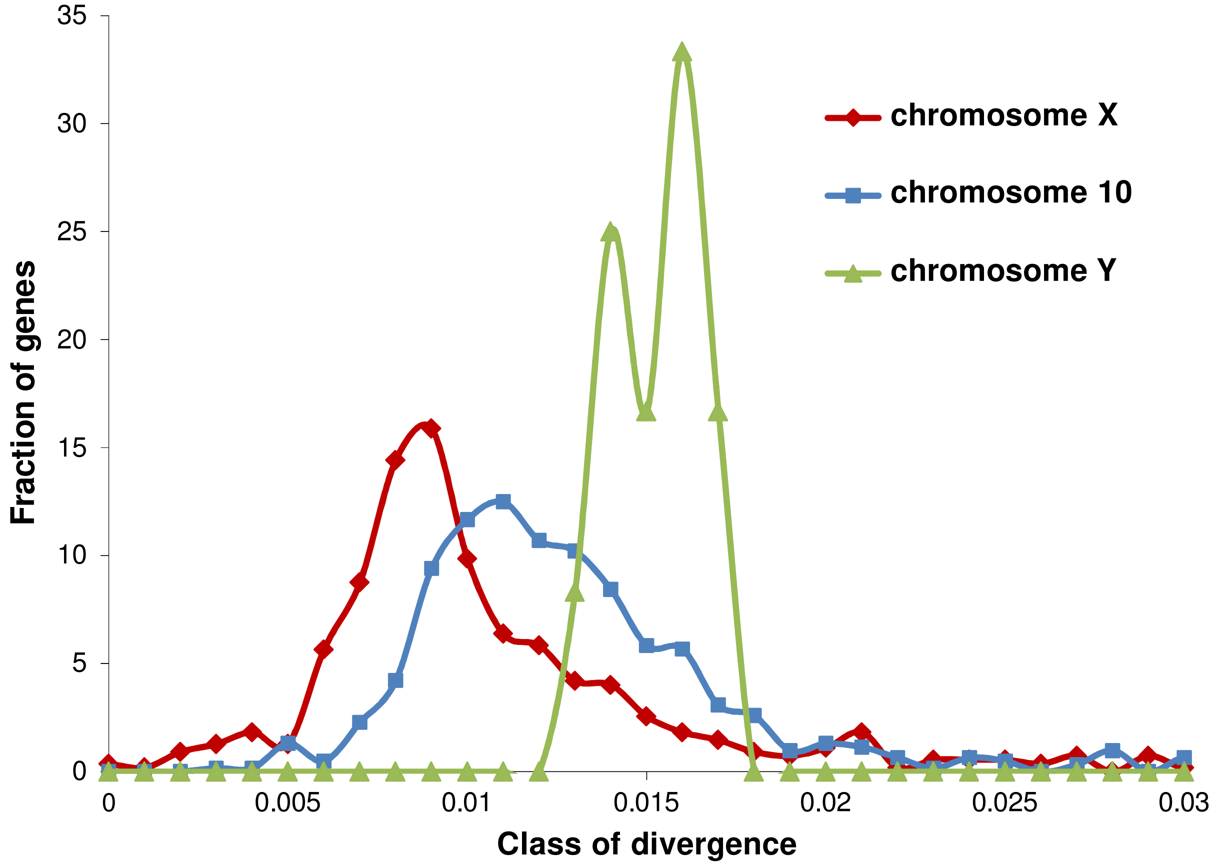

Figure S2. Results of computer simulations with faithful mating pairs for three assumptions on the recombination between X and Y chromosomes. The left panel (**a**, **c**, **e**). The ratio of the number of mutated alleles in the X chromosome to the 10th autosome (X/10) and in the Y chromosome to the X chromosome (Y/X), calculated for individuals in various age: new-borns, youth and adults. The right panel (**b**, **d**, **f**). The ratio of males to females (M/F) calculated for individuals in various age: new-borns, youths and adults. New-borns are individuals before the birth age; youths are individuals between the birth and reproductive age, and adults are individuals after the reproductive age.

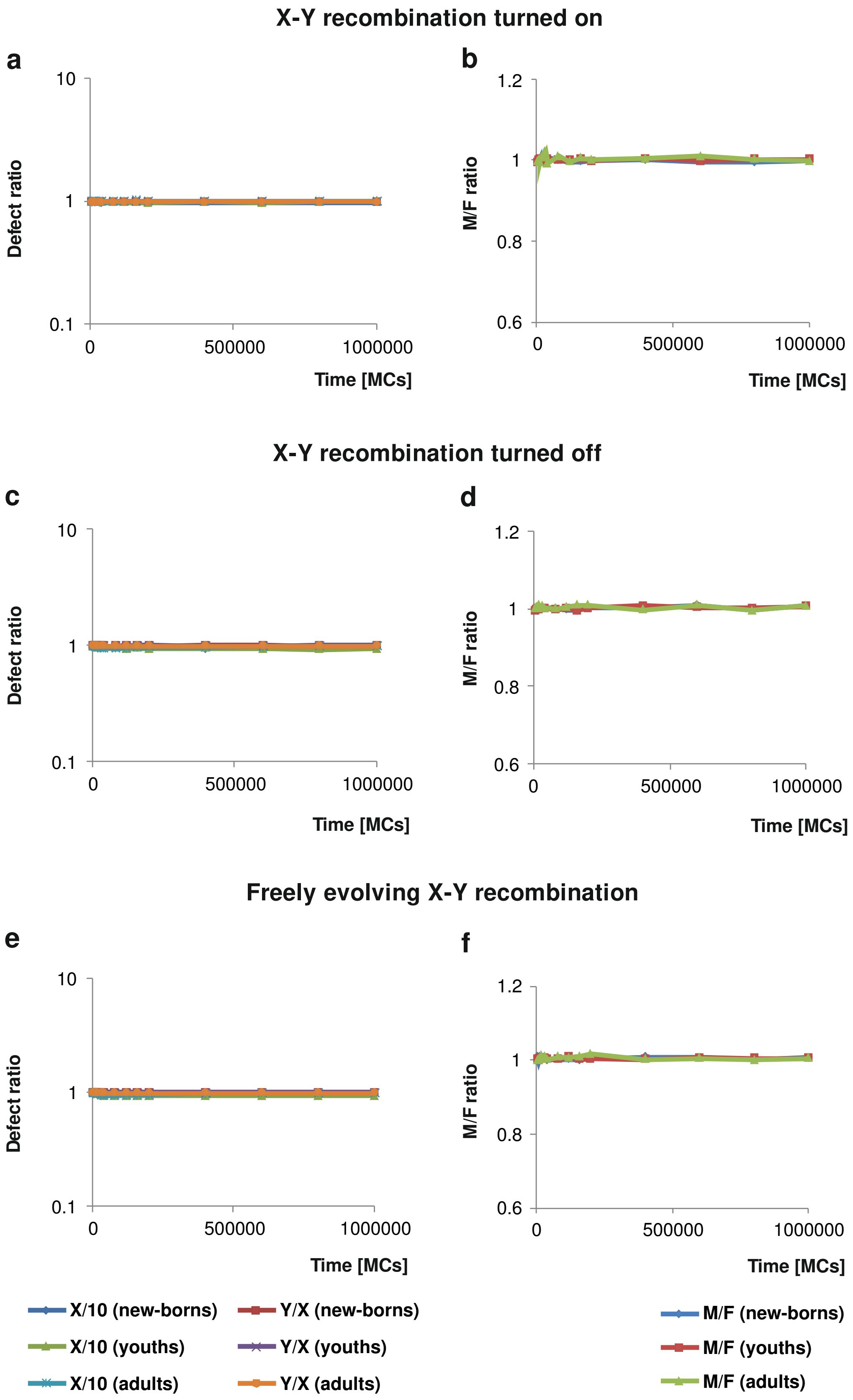

Figure S3. Fraction of defective alleles in the loci of the 10th autosome or sex chromosomes calculated in all individuals after 1,000,000 MCs assuming the limit of mutations responsible for genetic death T = 20. The x axis corresponds to genes ranked according to their activation time; the y axis corresponds to the mean fraction of deleterious alleles for the given gene; *b* is the time of birth; *R* is the first year of reproduction. The upper plots concern the simulations with turned on recombination between the X and Y chromosomes. Note, the growing fraction of defective loci activated after the minimum reproductive age. The bottom plots refer to the simulations with turned off recombination between the X and Y chromosomes. Note, the large fraction of defective loci on the Y chromosome independently on the activation time of genes.

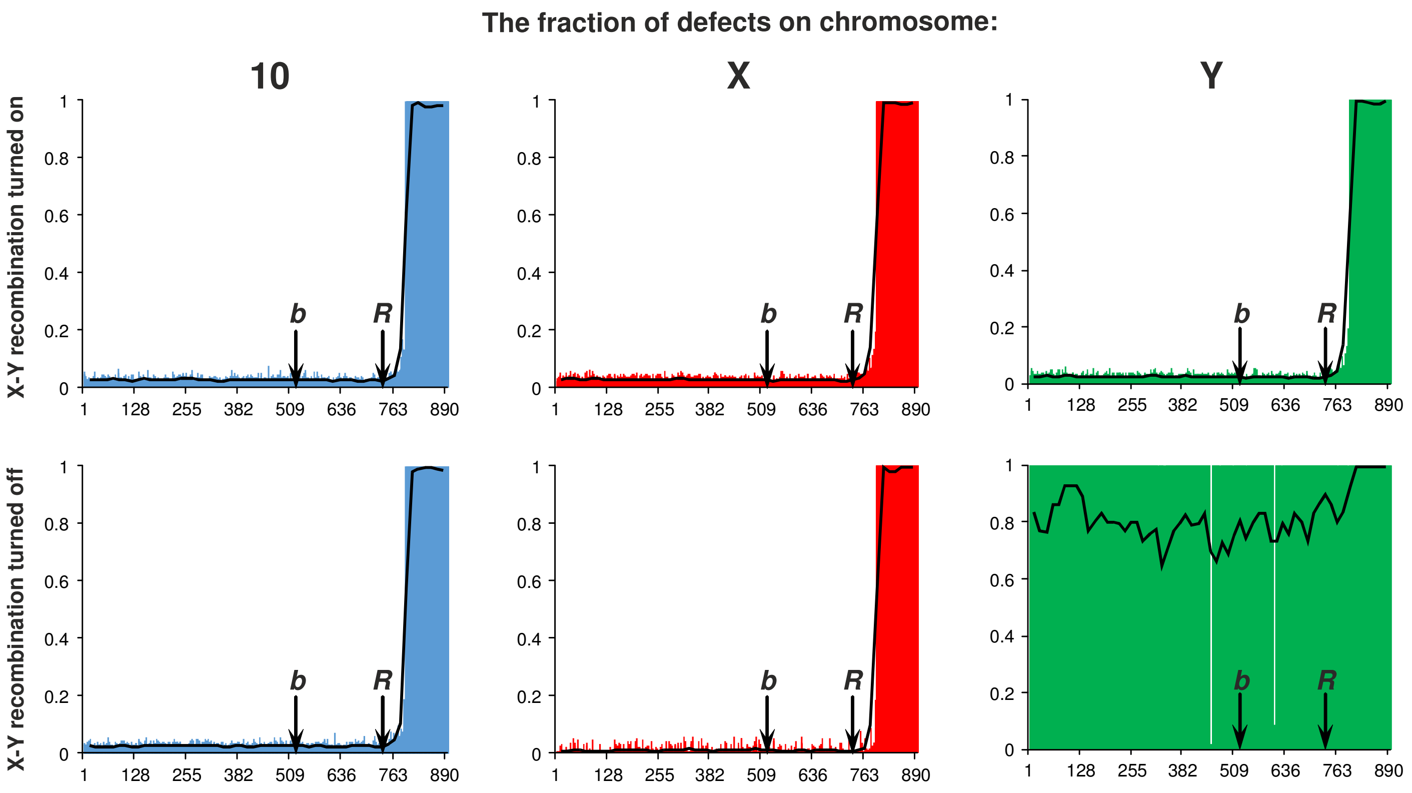

Figure S4. Results of computer simulations with unfaithful mating pairs for three assumptions on the recombination between X and Y chromosomes assuming the limit of mutations responsible for genetic death *T* = 20. The left panel (**a**, **c**, **e**). The ratio of the number of mutated alleles in the X chromosome to the 10th autosome (X/10) and in the Y chromosome to the X chromosome (Y/X), calculated for individuals in various age: new-borns, youths and adults. The right panel (**b**, **d**, **f**). The ratio of males to females (M/F) calculated for individuals in various age: new-borns, youths and adults. New-borns are individuals before the birth age; youths are individuals between the birth and reproductive age, and adults are individuals after the reproductive age. Points for the ratio of defective mutations in Y/X (new-borns) and Y/X (youths) as well as for X/10 (new-borns) and X/10 (youths) overlap.

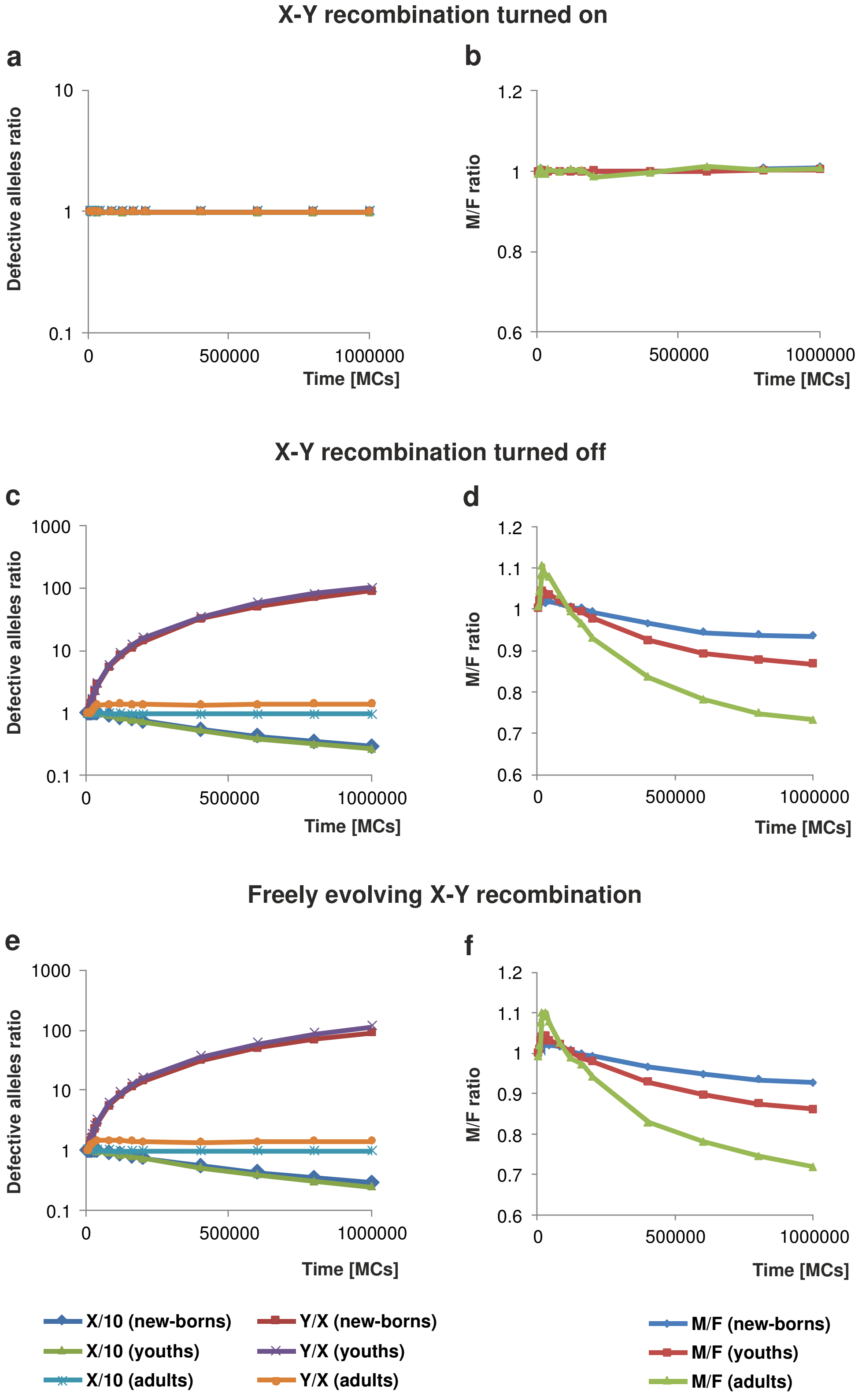

Figure S5. Changes in the recombination rate between the X and Y chromosomes during simulations for two reproductive strategies: unfaithful and faithful mating pairs, assuming the limit of mutations responsible for genetic death $T = 20$. Values on the y axis are mean of recombination in a given simulation step divided by the initial value typical of X chromosomes. The lattice assuming distance ranges for searching for a partner and putting a child was $D_p = 6$ and $D_c = 6$.

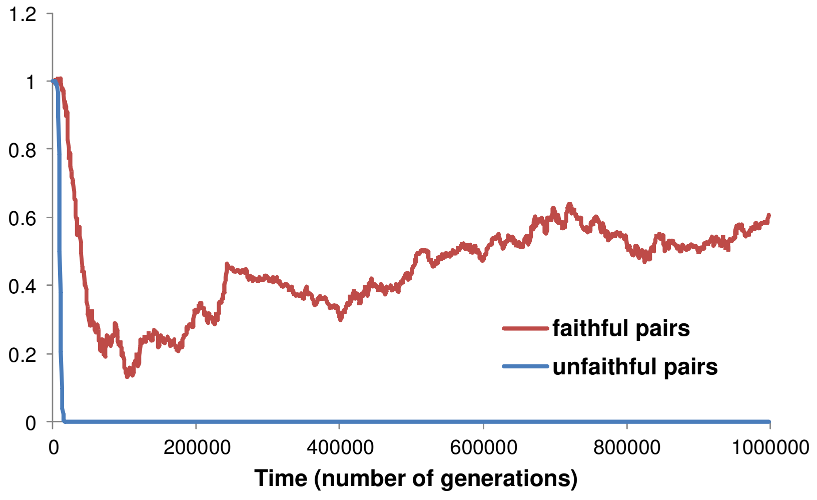

Supplement: Supplementary file 1 — Supplementary material [file 41598_2018_27219_MOESM1_ESM.pdf]
